# Incidence and mortality of community-acquired and nosocomial infections in Japan: a nationwide medical claims database study

**DOI:** 10.1186/s12879-024-09353-6

**Published:** 2024-05-23

**Authors:** Nozomi Takahashi, Taro Imaeda, Takehiko Oami, Toshikazu Abe, Nobuaki Shime, Kosaku Komiya, Hideki Kawamura, Yasuo Yamao, Kiyohide Fushimi, Taka‑aki Nakada

**Affiliations:** 1 Centre for Heart Lung Innovation, St. Paul’s Hospital, The University of British Columbia, 1081 Burrard Street, Vancouver, BC V6Z 1Y6 Canada; 2 Department of Emergency and Critical Care Medicine, Chiba University Graduate School of Medicine, Chiba, Japan; 3 Health Services Research and Development Center, University of Tsukuba, Tsukuba, Japan; 4 Department of Emergency and Critical Care Medicine, Tsukuba Memorial Hospital, Tsukuba, Japan; 5 Department of Emergency and Critical Care Medicine, Graduate School of Biomedical and Health Sciences, Hiroshima University, Hiroshima, Japan; 6 Ad Hoc Committee On Clinical Research Using DPC, The Japanese Association for Infectious Diseases, Tokyo, Japan; 7 Respiratory Medicine and Infectious Diseases, Faculty of Medicine, Oita University, Oita, Japan; 8 Department of Infection Control and Prevention, Kagoshima University Hospital, Kagoshima, Japan; 9 Department of Health Policy and Informatics, Tokyo Medical and Dental University Graduate School of Medical and Dental Sciences, Tokyo, Japan

**Keywords:** Infectious disease, Hospital-acquired infections, Aging, Sepsis, Organ dysfunction

## Abstract

**Background:**

It is important to determine the prevalence and prognosis of community-acquired infection (CAI) and nosocomial infection (NI) to develop treatment strategies and appropriate medical policies in aging society.

**Methods:**

Patients hospitalized between January 2010 and December 2019, for whom culture tests were performed and antibiotics were administered, were selected using a national claims-based database. The annual trends in incidence and in-hospital mortality were calculated and evaluated by dividing the patients into four age groups.

**Results:**

Of the 73,962,409 inpatients registered in the database, 9.7% and 4.7% had CAI and NI, respectively. These incidences tended to increase across the years in both the groups. Among the patients hospitalized with infectious diseases, there was a significant increase in patients aged ≥ 85 years (CAI: + 1.04%/year and NI: + 0.94%/year, *P* < 0.001), while there was a significant decrease in hospitalization of patients aged ≤ 64 years (CAI: -1.63%/year and NI: -0.94%/year, *P* < 0.001). In-hospital mortality was significantly higher in the NI than in the CAI group (CAI: 8.3%; NI: 14.5%, adjusted mean difference 4.7%). The NI group had higher organ support, medical cost per patient, and longer duration of hospital stay. A decreasing trend in mortality was observed in both the groups (CAI: -0.53%/year and NI: -0.72%/year, *P* < 0.001).

**Conclusion:**

The present analysis of a large Japanese claims database showed that NI is a significant burden on hospitalized patients in aging societies, emphasizing the need to address particularly on NI.

**Supplementary Information:**

The online version contains supplementary material available at 10.1186/s12879-024-09353-6.

## Background

Infectious diseases play an important role in hospital admissions, mortality, and medical costs worldwide [1, 2]. Among these, bacterial and fungal infections can cause sepsis, which is characterized by organ dysfunction and leads to a worse prognosis in these patients [3–5]. Several methods have been formulated to classify these infections for epidemiological investigation, including the pathogen, focus of infection, and setting of onset. According to setting of onset, infections can be divided into community-acquired infection (CAI) or nosocomial infection (NI) [6]. The setting of infection onset is crucial because these two settings differ in terms of pathogenic organs, type of infection, therapeutic strategy, and clinical course [7–9].

There have been several epidemiological studies on infectious diseases, including specific infectious sites or backgrounds, as well as individual pathogens, which have provided insight into the disease and its burden and informed future policy decisions [2, 10, 11]. However, few studies have reported the current situation and trends in the incidence and clinical outcomes of patients with bacterial and fungal infections from the perspective of onset of infection, despite many studies that have reported on sepsis showing increasing trends of incidence and worse clinical outcomes in patients with NI [12–14]. Considering the appropriate management of medical resources and decisions regarding healthcare policy, understanding the current situation of bacterial and fungal infections along with their trends of prevalence and prognosis in these two settings of infection onset should be considered important factors, along with other factors such as age and focus of infection [15]. In particular, since Japan has had the world's largest aging population since 2005, the original Japanese analysis could make an important contribution to future healthcare policy in a country with aging population.

The aim of this study was to describe the epidemiology of the incidence and characteristics of CAI and NI, and to verify the hypothesis that NI has a higher mortality rate than CAI in Japan using data from the national claims-based database, which has more than 70 million inpatients.

## Methods

### Study design and data source

We conducted a retrospective observational study using the Japanese Diagnosis Procedure Combination (DPC) system, which consists of large administrative claims data on reimbursement, covering more than 80% of acute care hospitals [16, 17]. This DPC system contains two types of codes, diagnostic and procedure codes, in addition to basic information such as age, sex, and patient outcomes. Each required code is registered for each hospitalized patient on a daily basis. The International Classification of Diseases, 10th Revision (ICD-10) codes were used as the diagnostic code, in addition to the name of the disease that was the main cause of hospitalization, comorbidities at the time of admission, and new names of diseases added during hospitalization, which were registered with up to six codes on admission and four codes after admission. The procedure code was originally defined in Japanese, and all procedure devices needed during hospitalization, including organ support or drugs used regardless of the route of administration, were coded and registered [18, 19]. Data of patients admitted between January 2011 and December 2019 were extracted from the DPC database. This study was approved by the Institutional Review Board of Chiba University Graduate School of Medicine and was performed in accordance with the tenets of Declaration of Helsinki. The need for informed consent was waived according to the review board (approval number, 3429).

### Definition and data collection

All inpatient datasets with codes for both culture tests and antibiotic administration via intravenous infusion were extracted from the DPC database. CAI group was defined as the group of patients who received antibiotics within two days of hospitalization, and NI group was defined as the group of patients who received antibiotics after the third day of hospitalization. To exclude prophylactic administration of antibiotics, we extracted those who received antibiotics for ≥ 3 days, but we also included patients who received antibiotics for < 3 days in the context of transferred and deceased patients. Accordingly, the CAI group satisfied the following conditions: (i) antibiotic administration commenced within two days of hospitalization and continued for at least four days, or (ii) antibiotics were started within two days of hospitalization and continued for less than four days, and the outcome was death or transfer. The NI group satisfied the following conditions: (i) antibiotic administration commenced at least three days after hospitalization and continued for at least four days, or (ii) antibiotics were started at least three days after hospitalization and continued for less than four days, and the outcome was death or transfer.

Basic patient characteristics, including age, sex, along with admission and discharge dates, were available in DPC. Whether or not the patients were admitted to the ICU could be referenced from the procedure code. Comorbidities and focus of infection were classified and extracted using ICD-10 codes (Supplementally file; Table S1, S2). Comorbidities included malignant tumor, hypertension, diabetes mellitus, heart failure, cerebrovascular disease, chronic respiratory disease, ischemic heart disease, and chronic renal failure. According to the current definition of sepsis, we defined sepsis using a combination of ICD-10 and procedure codes that indicated acute organ dysfunction, as previously reported [20] (Supplementally file; Table S3). Organ supportive therapy was investigated using procedure codes, including oxygen therapy, mechanical ventilation, vasopressor use, or renal replacement therapy. The duration of administration of antibiotics agents and therapeutic drugs was extracted from the procedure codes. Antibiotics agents were based on the international classification [21]. It should be noted that organ dysfunction and organ supportive therapy were extracted during the whole period of hospitalization, and the contribution of infection to length of hospitalization and cost was unknown. Therefore, the causal association to the corresponding infectious disease was unknown and should be interpreted as indicative of the nature of each patient group.

Medical cost in Japanese yen, which was calculated as the summary of medical fees including medical procedure, fee of drugs, or medical material cost, was converted into U.S. dollars in accordance with the exchange rate on January 14, 2023 (127 yen = $1 USD).

### Statistical analysis

The primary outcomes were annual changes in hospitalization and mortality rates comparing CAI and NI. Secondary outcomes were in-hospital mortality in all investigated years, ICU admission, organ support, duration of initial antibiotics and all antibiotics (days), length of hospital stay, and medical cost. Primary outcomes were analyzed in the four age groups according to the criteria previously reported for older adult patients [22, 23]. Hospitalization rates were analyzed separately for each focus of infection in a subgroup analysis. Patients with repeat hospitalizations were excluded from the analysis of in-hospital mortality for accurate analysis of the factors associated with death. Comorbidities were scored using the Elixhauser comorbidity scores for mortality analysis [24, 25].

Cochran-Armitage test was used for 10-year trend analyses of CAI and NI prevalence relative to total hospitalizations and in-hospital mortality. Analysis of covariance was used to test the difference in the slope of linear regression analysis of the 10-year trend between the two groups obtained using the least-squares method.

Associations between CAI or NI and the outcomes were analyzed using a Poisson regression generalized linear mixed-effects model adjusted for patient age, sex, and Elixhauser comorbidity scores. Unadjusted and adjusted differences in outcomes were reported with 95% confidence intervals (CIs).

Continuous variables were presented as medians with interquartile ranges. Categorical variables were presented as numbers and percentages. Statistical significance was set at *P* < 0.05. Analyses were performed using R version 4.1.2 (R Foundation for Statistical Computing, Vienna, Austria, http://www.R-roject.org/) and PRISM version 8 (GraphPad Software, Inc., La Jolla, California, USA).

## Results

**Fig. 1:**
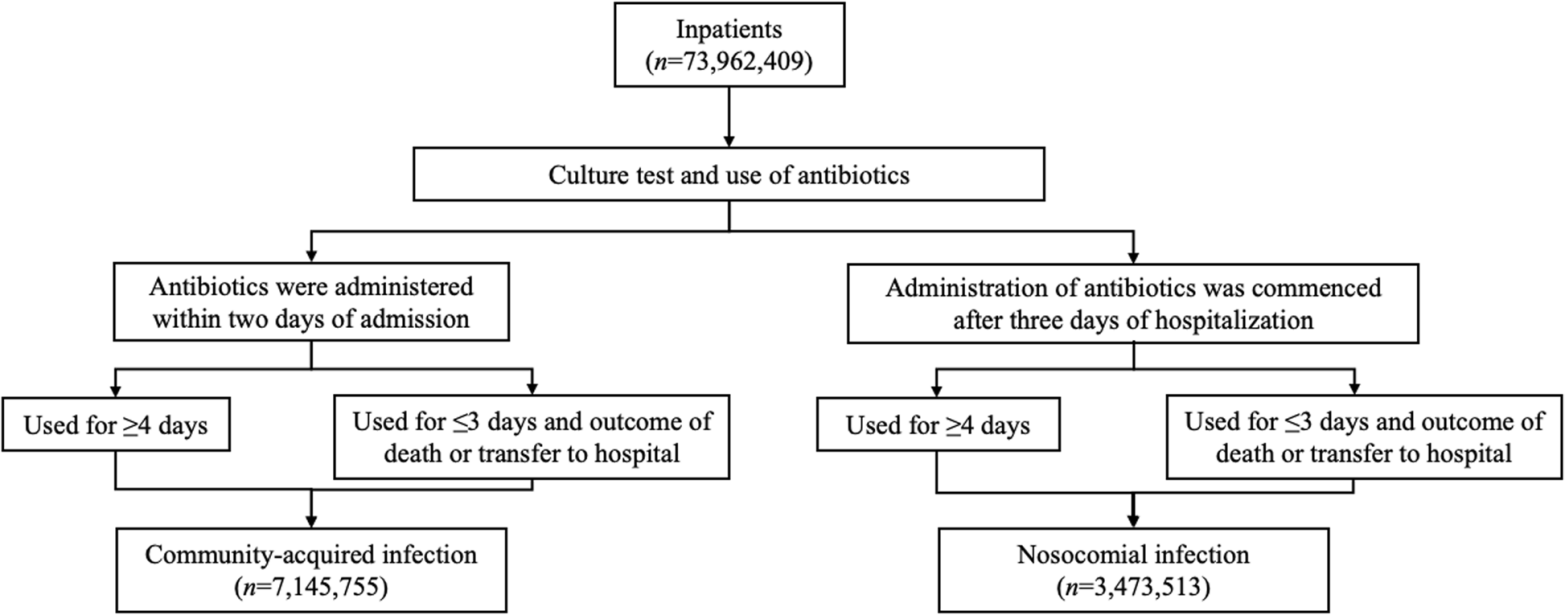
Flow chart demonstrating the selection of study population

Of the 73,962,409 hospitalized cases recorded between 2010–2019, a total of 7,145,755 (9.7%) and 3,473,513 (4.7%) patients met the definitions of CAI and NI, respectively (Fig. 1).

Regarding the baseline characteristics, patients with CAI were significantly younger (CAI: 74 years and NI: 75 years, *P* < 0.001) and comprised lower proportion of males (CAI: 3,965,319 patients [55.5%] and NI: 2,010,875 patients [57.9%], *P* < 0.001) in comparison to than that of patients with NI (Table 1). The proportion of comorbidities was lower in the CAI group than in the NI group for all factors except chronic pulmonary disease (CAI: 737,483 patients [10.3%] and NI: 247,372 patients [7.1%], *P* < 0.001). Respiratory infection was the most common focus of infection in the CAI group (2,447,113 patients [34.2%]), whereas abdominal infection was the most common infection in the NI group (923,357 patients [26.6%]). The rate of sepsis was nearly twice as high in the NI group (CAI: 784,123 patients [11.0%] and NI: 692,266 patients [19.9%], *P* < 0.001). Moreover, organ dysfunction was more common in the NI group in comparison to the CAI group (CAI:1,048,022 patients [14.7%] and NI: 814,034 patients [23.4%], *P* < 0.001). The duration of administration of initial and all antibiotics were longer in the NI group than in the CAI group (initial antibiotics, CAI: 4 days and NI: 7 days, *P* < 0.001; all antibiotics, CAI: 6 days and NI: 12 days, *P* < 0.001).

**Table 1:** Clinical characteristics of patients with infections

Characteristic	Community-acquired infections *n* = 7,145,755	Nosocomial infections *n* = 3,473,513
Age, yr	74 (51–84)	75 (63–84)
Male, n (%)	3,965,319 (55.5)	2,010,875 (57.9)
Comorbidities, n (%)		
Diabetes mellitus	1,190,434 (16.7)	695,748 (20.0)
Solid cancer	1,074,732 (15.0)	763,688 (22.0)
Congestive heart failure	927,740 (13.0)	572,944 (16.5)
Cerebrovascular disease	836,262 (11.7)	559,488 (16.1)
Chronic pulmonary disease	737,483 (10.3)	247,372 (7.1)
Non-solid cancer	391,759 (5.5)	438,367 (12.6)
Liver disease	352,641 (4.9)	226,006 (6.5)
Chronic renal failure	285,979 (4.0)	188,147 (5.4)
Focus of infection, n (%)		
Respiratory	2,447,113 (34.2)	923,357 (26.6)
Abdominal	1,815,670 (25.4)	976,521 (28.1)
Genitourinary	1,035,432 (14.5)	494,329 (14.2)
Skin and soft tissue	360,863 (5.1)	163,922 (4.7)
Central nervous system	146,591 (2.1)	76,583 (2.2)
Endocarditis/Circulatory	82,143 (1.1)	62,592 (1.8)
Sepsis, n (%)	784,123 (11.0)	692,266 (19.9)
Organ dysfunction, n (%)	1,048,022 (14.7)	814,034 (23.4)
Respiratory	676,885 (9.5)	554,017 (15.9)
Cardiovascular	477,888 (6.7)	369,460 (10.6)
Renal	175,686 (2.5)	165,778 (4.8)
Hematological	95,287 (1.3)	111,354 (3.2)
Neurologic	16,327 (0.2)	10,879 (0.3)
Hepatic	12,251 (0.2)	8,486 (0.2)
Duration of initial antibiotics, days	4 (6–9)	7 (5–11)
Duration of all antibiotics, days	6 (8–13)	12 (7–20)

All characteristics differed significantly between community-acquired and nosocomial infections (*P* < 0.001)

Data are described as median and interquartile range, *ICU* Intensive care unit

**Fig. 2:**
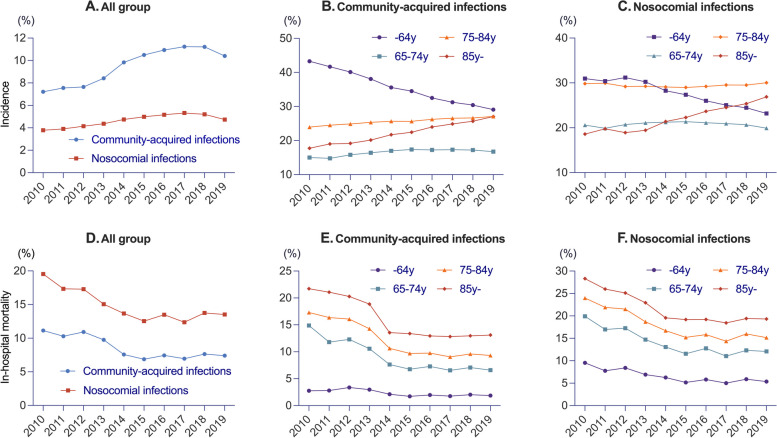
Annual changes in hospitalization and in-hospital mortality by infectious disease. **A **Incidence of hospitalization across all inpatients (community acquired infection: + 0.49%/year [95%CI; 0.31%–0.67%], adjusted *R*^2^ = 0.81, *P* < 0.001 and nosocomial infection: + 0.15%/year [95%CI; 0.08%–0.23%], adjusted *R*^2^ = 0.71, *P* = 0.001); **B **Proportion of hospitalization in community-acquired infections by age subgroups (≤ 64 years: -1.63%/year [95%CI; -1.77%– -1.48%], adjusted *R*^2^ = 0.99, *P* < 0.001; 65–74 years: + 0.26%/year [95%CI; 0.11%–0.41%], adjusted *R*^2^ = 0.63, *P* = 0.0037; 75–84 years: + 0.33%/year [95%CI; 0.29%–0.37%], adjusted *R*^2^ = 0.97, *P* < 0.001; ≥ 85 years: + 1.04%/year [95%CI; 0.96%–1.12%], adjusted *R*^2^ = 0.99, *P* < 0.001); **C **Proportion of hospitalization in nosocomial infections by age subgroups (≤ 64 years: -0.95%/year [95%CI; -1.13%– -0.76%], adjusted *R*^2^ = 0.94, *P* < 0.001; 65–74 years: *P* = 0.95; 75–84 years: *P* = 0.99; ≥ 85 years: + 0.94%/year [95%CI; 0.76%–1.12%], adjusted *R*^2^ = 0.94, *P* < 0.001); **D **In-hospital mortality of community-acquired and nosocomial infection (community acquired infection: -0.53%/year [95%CI; -0.79%– -0.27%], adjusted *R*^2^ = 0.71, *P* = 0.0015 and nosocomial infection: -0.72%/year [95%CI; -1.05%– -0.38%], adjusted *R*^2^ = 0.72, *P* = 0.0011); **E **Community-acquired infections by age subgroups (≤ 64 years: -0.15%/year [95%CI; -0.25%– -0.05%], adjusted *R*^2^ = 0.56, *P* = 0.075; 65–74 years: -0.89%/year [95%CI; -1.24%– -0.54%], adjusted *R*^2^ = 0.79, *P* < 0.001; 75–84 years: -1.02%/year [95%CI; -1.38%– -0.66%], adjusted *R*^2^ = 0.82, *P* < 0.001; ≥ 85 years: -1.15%/year [95%CI; -1.61%– -0.69%], adjusted *R*^2^ = 0.78, *P* < 0.001); **F **Nosocomial infections by age subgroups (≤ 64 years: -0.44%/year [95%CI; -0.64%– -0.24%], adjusted *R*^2^ = 0.73, *P* = 0.001; 65–74 years: -0.86%/year [95%CI; -1.23%– -0.48%], adjusted *R*^2^ = 0.75, *P* = 0.001; 75–84 years: -1.01%/year [95%CI; -1.40%– -0.62%], adjusted *R*^2^ = 0.79, *P* < 0.001; ≥ 85 years: -1.04%/year [95%CI; -1.48%– -0.60%], adjusted *R*.^2^ = 0.76, *P* < 0.001)

The annual proportion of patients with CAI and NI across inpatients significantly increased over the years (CAI: + 0.49%/year, *P* < 0.001; NI: + 0.15%/year, *P* = 0.001), and the difference in trends between the two groups was significant (*P* = 0.0012) (Fig. 2A). Among the patients hospitalized with infectious diseases, in both groups, there was a significant decrease in hospitalization in patients aged ≤ 64 years (CAI: -1.63%/year, *P* < 0.001 and NI: -0.94%/year, *P* < 0.001), while there was a significant increase in patients aged ≥ 85 years (CAI: + 1.04%/year, *P* < 0.001; NI: + 0.94%/year, *P* < 0.001) (Fig. 2B, C). Patients aged ≥ 65 years and < 75 years, and aged ≥ 75 years and < 85 showed significant increase in only CAI. This trend was also observed when the ≥ 75 years group was integrated (Supplementary file; Figure S1). In terms of trends by focus of infection, respiration decreased significantly in CAI but not in NI (CAI: -0.57%/year, *P* = 0.0031 and NI: *P* = 0.081), and genitourinary infection increased significantly in both groups (CAI: + 0.46%/year, *P* < 0.001 and NI: + 0.45%/year, *P* < 0.001) (Supplementary file; Figure S2).

The 30-day mortality and in-hospital mortality was significantly higher in the NI group than in the CAI group after adjusting for age, sex, and Elixhauser comorbidity score (30-day mortality: unadjusted mean difference, 1.3% [95%CI, 1.2–1.3]; adjusted mean difference, 1.0% [95%CI; 1.0–1.1], in-hospital mortality: unadjusted mean difference, 6.2% [95%CI, 6.2–6.3]; adjusted mean difference, 4.7% [95%CI; 4.7–4.7]) (Table 2). Even after excluding transfers, both 30-day mortality (CA; 259,993/4,229,528patisnts [6.1%], NI; 151,470/1,717,715patients [8.8%]) and in-hospital mortality (CA; 413,513/4,229,528patisnts [9.8%], NI; 336,805/1,717,715patients [19.6%]) were both significantly higher in the NI group (*P* < 0.001). The ICU admission rate was significantly higher in the NI group (unadjusted mean difference, 3.0%; adjusted mean difference, 2.3%), but it should be noted that the causal relationship between ICU admission and infection and the timing of onset of infection were unknown in this analysis. The annual in-hospital mortality significantly decreased in both CAI and NI (CAI: -0.53%/year, *P* = 0.0015 and NI: -0.72%/year, *P* = 0.0011) (Fig. 2D). In terms of age group, the in-hospital mortality for both CAI and NI significantly decreased in the four age groups: ≥ 65 and < 75 years, ≥ 75 and < 85 years, and ≥ 85 years (Fig. 2E, F, Supplementary file; Figure S1). NI had a higher in-hospital mortality rate than CAI for all infection foci (*P* < 0.001) (Table 3). In each infection focus, respiratory infections had the highest mortality in both groups (CAI: 154,566 patients [8.8%]; NI: 115,117 patients [18.4%]), followed by endocarditis/circulatory infections in CAI (4,417 patients [7.9%]) and abdominal infections in NI (86,669 patients [13.4%]). Liver dysfunction was associated with the highest mortality for both CAI and NI (CAI: 3,072 patients [32.3%]; NI: 2,679 patients [41.7%]), and all organ dysfunctions, except hematological dysfunction, were associated with significantly higher mortality for NI (*P* < 0.001) (Table 3). The central nervous system mortality rate did not decrease in either group (CAI, *P* = 0.38; NI, *P* = 0.062) (Supplementary file; Figure S3).

**Table 2:** Outcomes of community-acquired and nosocomial infections

	Community-acquired infections (*n* = 5,012,428)	Nosocomial infections (*n* = 2,327,195)	Un-adjusted difference (95% CI)	Adjusted difference (95% CI)
30-day mortality, n (%)	260,006 (5.2)	151,478 (6.5)	1.3 (1.2–1.3)	1.0 (1.0–1.1)
In-hospital mortality, n (%)	413,539 (8.3)	336,826 (14.5)	6.2 (6.2–6.3)	4.7 (4.7–4.7)
ICU admission, n (%)	408,831 (8.2)	259,517 (11.2)	3.0 (2.9–3.0)	2.3 (2.2–2.3)
Organ support, n (%)	2,678,440 (53.4)	1,588,878 (68.3)	14.8 (14.8–14.9)	11.5 (11.5–11.6)
Oxygen therapy	2,443,744 (48.8)	1,445,358 (62.1)	13.4 (13.3–13.4)	9.3 (9.2–9.4)
Mechanical ventilation	521,122 (10.4)	425,624 (18.3)	7.9 (7.8–7.9)	8.3 (8.2–8.3)
Vasopressor	365,276 (7.3)	273,622 (11.8)	4.5 (4.4–4.5)	4.7 (4.7–4.7)
Renal replacement therapy	118,344 (2.3)	114,194 (4.9)	2.5 (2.5–2.6)	2.3 (2.3–2.4)
Hospital length of stay, day	25 (37)	49 (130)	23 (23–24)	23 (22–23)
Medical cost per patients, $	10,336 (18,260)	19,547 (27,585)	9,210 (9,171–9,250)	9,209 (9,174–9,242)

Continuous variables are presented as mean (S.D.), and adjusted by age, sex and Elixhauser comorbidity score

*ICU* Intensive care unit, *CI* Confidence interval

**Table 3:** Mortality in terms of infectious focus and organ dysfunction

	Community-acquired infections	Nosocomial infections	Un-adjusted odds ratio (95% CI), *P*-value	Adjusted odds ratio (95% CI), *P*-value
Infectious focus, n (%)				
Respiratory	154,566 (8.8)	115,177 (18.4)	2.33 (2.30–2.34), < 0.001	1.81 (1.80–1.83), < 0.001
Abdominal	94,373 (7.6)	86,669 (13.4)	1.90 (1.88–1.92), < 0.001	1.81 (1.79–1.82), < 0.001
Genitourinary	41,544 (5.9)	36,105 (10.7)	1.91 (1.88–1.94), < 0.001	1.77 (1.74–1.80), < 0.001
Skin and soft tissue	10,898 (4.0)	9,541 (7.9)	2.06 (2.00–2.12), < 0.001	1.74 (1.69–1.79), < 0.001
Central nervous system	5,864 (4.8)	5,471 (8.9)	1.95 (1.87–2.02), < 0.001	1.36 (1.30–1.41), < 0.001
Endocarditis/Circulatory	4,417 (7.9)	4,530 (10.6)	1.37 (1.31–1.43), < 0.001	1.39 (1.32–1.44), < 0.001
Organ dysfunction, n (%)				
Respiratory	132,604 (25.4)	111,551 (26.2)	1.04 (1.03–1.05), < 0.001	1.03 (1.02–1.04), < 0.001
Cardiovascular	81,684 (22.4)	71,406 (26.1)	1.23 (1.21–1.24), < 0.001	1.16 (1.15–1.18), < 0.001
Renal	37,816 (30.0)	37,279 (32.0)	1.10 (1.08–1.12), < 0.001	1.08 (1.06–1.10), < 0.001
Hematological	16,524 (27.1)	16,587 (27.4)	1.01 (0.99–1.04), 0.32	1.02 (1.00–1.05), 0.078
Neurologic	1,119 (8.7)	1,104 (13.5)	1.63 (1.49–1.78), < 0.001	1.33 (1.22–1.46), < 0.001
Hepatic	3,072 (32.3)	2,679 (41.7)	1.50 (1.40–1.60), < 0.001	1.47 (1.37–1.58), < 0.001

Continuous variables are presented as mean (S.D.), and adjusted by age, sex and Elixhauser comorbidity score

*CI* Confidence interval

Furthermore, NI had higher organ support (unadjusted mean difference 3.0% [95%CI; 2.9–3.0]; adjusted mean difference 2.3% [95%CI; 2.2–2.3]), medical cost per patient (unadjusted mean difference $9,210 [95%CI; 9,171–9,250]; adjusted mean difference $9,209 [95%CI; 9,147–9,242]), and longer duration of hospital stay (unadjusted mean difference 23 days [95%CI; 23–24]; adjusted mean difference 23 days [95%CI; 22–23]).

## Discussion

The present analysis of a large Japanese claims database consisting of more than 10 million patients showed that the incidences of both CAI and NI tended to increase across the years especially in patients aged ≥ 85 years. Moreover, in-hospital mortality was found to be significantly higher in the NI group compared to the CAI group, while there was a decreasing trend of mortality in both groups.

CAI and NI differ in background disease, cause, treatment including antimicrobial therapy, and prevention, which indicates the importance of understanding the characteristics, prevalence, and mortality of each　 [26, 27]. The present study is the most extensive epidemiological study on the incidence and mortality of CAI and NI worldwide till date, and at the same time, the most comprehensive nationwide study conducted in Japan. The median age of the study population was approximately 75 years, which indicates that Japan is the most aged country in the world. Therefore, these results suggest the importance of future healthcare policies for older adults and nosocomial infections in an aging society.

Our results indicate that the incidence of both infections increased. Few studies have reported changes in the prevalence of CAI and NI, although some have only reported the prevalence of NI. A 2010 Centers for Disease Control and Prevention (CDC) prevalence study of 183 hospitals in the US reported that approximately 4% of hospitalized patients had NI, which is in concordance with our findings [28]. Furthermore, a previous study that conducted surveys in 2002 and 2009 to measure the prevalence and characteristics of NI in Canadian hospitals showed an 11.7% increase in the prevalence of NI, with the increment being maximum in terms of urinary tract infections during this period [29]. Additionally, a meta-analysis of the global prevalence of NI from 2000–2021 reported an annual increase of 0.06% [30]. This trend is similar to our findings, suggesting that the incidence of NI has been increasing despite appropriate infection prevalence. However, our results showed a decrease in the last year of data collection, suggesting that the prevalence may decrease further in the future. However, estimating changes in hospitalizations due to infectious diseases after 2020 is an arduous task as COVID-19 changed the landscape of hospitalizations due to infectious diseases [31]. It should be noted that the incidence of patients aged ≥ 85 years, called the oldest-old, among those hospitalized for infectious diseases clearly increased in both groups (1.04%/year for CAI, 0.94%/year for NI). Statistics in Japan shows that the population aged ≥ 85 years increased from 3.8 million in 2010 to 6.2 million in 2020 (6.3%/year increase) [32], which may reflect the aging of Japanese population.

In-hospital mortality was nearly twice as high in the NI group as in the CAI group regardless of the focus of infection, whereas the incidence was twice as high in the CAI group in comparison to the NI group. Previous studies on sepsis have also shown that the mortality rate for NI was higher than that for CAI, and Japanese studies have reported the same two-fold increase [12–14, 33–35]. A Canadian study in 2017 showed that the overall mortality rate among patients with at least one nosocomial infection was 16.6%, which is similar to our finding of 14.5% [36]. Some studies have suggested that the underlying conditions of patients, including immunosuppression, type and severity of the infection, in-hospital interventions, and increased bacteremia-induced septic shock, are associated with worse outcomes in NI [37]. In our study, comorbidities excluding chronic lung disease were significantly higher, and sepsis was approximately twice as common in the NI group, which may have contributed to the high mortality rate. In addition, ICU admission and organ support were significantly higher in the NI group after adjustment for other factors, reflecting the higher severity of NI compared to CAI, indicating that NI consumes more medical resources and creates a burden. Conversely, the incidence increased in both groups, but the mortality rate decreased over time. This may be due to the fact that the incidence of respiratory infections, which have a high mortality rate, has decreased, whereas the incidence of genitourinary infections, which have a low mortality rate, has increased. Mortality rates decreased for each infection, especially for abdominal infections. This could be due to appropriate antimicrobial stewardship and decrease in the number of antimicrobial-resistant organisms as a result of the development of guidelines for specific focus or sepsis, which are associated with high mortality and spread of antimicrobial stewardship in clinical practice [38, 39]. In Japan, routine pneumococcal vaccination of elderly people aged 65 years or older and patients at high risk of the infectious disease began in 2014. In the same year, the Japanese government issued a ministerial ordinance directing nosocomial infection control measures, which became an additional target for the medical reimbursement. These medical policies may have contributed to the change in the mortality rate. On the other hand, the incidence continues to increase, especially among the elderly, suggesting the need to implement medical policies to prevent both community-acquired and nosocomial infections, with particular emphasis on the elderly aged 75 years or older. This study may serve as a basis for publicizing such policies. However, this study did not clearly identify the causes of mortality, and future research on related factors is warranted. Furthermore, it should be noted that linear regression may not necessarily be optimal for 10-year trends in incidence and mortality of the present study. Although the Cochran-Armitage test, which is a pre-defined method for trend analysis, uses a regression line, the actual annual proportion of patients with CAI and NI across inpatients may have peaked from 2017 to 2018 and then started to decline. Also, the in-hospital mortality for both CAI and NI may have changed since around 2014. Further long-term data accumulation would be helpful to establish appropriate analytical methods and models, and the trend results should be interpreted with caution.

The present study had a few limitations. First, this study includes only some bacterial and fungal infections in the broad sense of the term, and does not include viral or special infections, limiting information on treatment methods. Furthermore, the database does not include information on the microorganisms causing the infection or the culture results, so it is uncertain whether they were truly causing the infection. Therefore, the potential selection bias occurred in the selection of infections, and is an issue in research design and methodology, while these findings may contribute useful information for the interpretation of prognosis, as NI is expected to be associated with a higher proportion of bacteria that are resistant to antimicrobial agents. Second, the last year of data used in this analysis was just before the COVID-19 pandemic and did not reflect COVID-19, which continues to have a significant impact worldwide. The impact of COVID-19 on community-acquired pneumonia should be considered in future data collection and analysis as it may have caused particularly large changes in CAI. Third, patients who were administered antimicrobials within two days of transfer were included in the CAI group. We were unable to distinguish this because it was not possible to refer to the pretransfer records in conjunction with each other. Fourth, some nosocomial infections, such as catheter-related and surgical site infections, were not categorized as the focus of infection in this study. This is because ICD-10 defines T81.4 as 'Infection following a procedure, not elsewhere classified', which may include these diseases and other abscesses regardless of site. Similarly, T82.7 is defined as 'Infection and inflammatory reaction due to other cardiac and vascular devices, implants, and grafts', was not included in the focus of infection since it may include infections such as catheter and surgical site infections. It is difficult to completely isolate the focus of infection using only the ICD-10 codes, which is a limitation of the method used in this study. Fifth, the results found on the incidence and mortality rate of CAI and NI might be specific to the Japanese medical environment including medical insurance system and the access to healthcare, which leads that these are not necessarily applicable to other regions or countries. Therefore, it is important to adapt the results to each local health care policy and situation. Sixth, although this study included more than 80% hospitals in Japan that were members of the DPC system, the number of eligible hospitals changed over the 10-year period, showing an increasing trend (Supplementary file; Table S4). Hospitals with a large number of beds did not show an increasing trend, while hospitals with a small number of beds showed an increasing trend. Therefore, it should be noted that the decrease in mortality in particular may have reflected an increase in the proportion of patients with non-severe illnesses included, which has limitations in its interpretation.

## Conclusions

Japanese claims database of more than 10 million patients revealed an increase in trends of hospitalization in both CAI and NI groups especially in patients aged ≥ 85 years and a significant burden in hospitalized patients with NI in the aging societies. In-hospital mortality was significantly higher in the NI group than that in the CAI group, whereas there was a decreasing trend in mortality in both the groups.

### Supplementary Information

**Supplementary Material 1.**

## Data Availability

The datasets used during the study are available from the corresponding author on reasonable request.

## Abbreviations

CAI: Community-acquired infection
NI: Nosocomial infection
ICU: Intensive care unit
DPC: Diagnosis procedure combination
ICD-10: International statistical classification of diseases and related health problems 10th revision
CI: Confidence interval
SOFA: Sequential organ failure assessment
