# Supplementary material for: Incidence and mortality of community-acquired and nosocomial infections in Japan: a nationwide medical claims database study
Source: BMC Infect Dis. 2024 May 23;24:518. doi: 10.1186/s12879-024-09353-6 (PMC11112762; doi:10.1186/s12879-024-09353-6)

Supplementary file

Incidence and mortality of community-acquired and nosocomial infections in Japan: A nationwide medical claims database study.

Nozomi Takahashi MD PhD[1,2], Taro Imaeda MD PhD[2], Takehiko Oami MD PhD[2], Toshikazu Abe MD MPH PhD[3,4], Nobuaki Shime MD PhD[5,6], Kosaku Komiya MD MPH PhD[6,7], Hideki Kawamura MD PhD[6,8], Yasuo Yamao[2], Kiyohide Fushimi MD PhD[9], Taka-aki Nakada MD PhD[2,6]

Table S1. ICD-10 codes for focus of infection.
Table S2. Codes for focus of comorbidities.
Table S3. ICD-10 and Japanese procedure claims codes for organ dysfunction.
Table S4. Changes in the number of hospitals subject to DPC system
Figure S1. Annual changes in hospitalization by infectious disease and in-hospital mortality.
Figure S2. Proportion of focus in hospitalized patients by infection.
Figure S3. Trends in in-hospital mortality on each focus

Table S1. ICD-10 codes for focus of infection

Respiratory	A15, A16, A420, A430, A510, B371, B380, B381, B382, B390, B391, B392, B400, B401, B402, B410, B440, B441, B450, B460, J13, J14, J15, J16, J17, J20
Abdominal	A00, A01, A02, A03, A04, A05, A06, A07, A08, A09, A183, A421, A511, A546, A563, B462, K35, K56, K57, K60, K61, K63, K65, K66, K67, K750, K80, K81, K82, K83, K85
Genitourinary	A181, A540, A541, A560, A562, A590, B373, B374, N080, N13, N151, N16, N20, N21, N290, N291, N30, N33, N34, N390, N41, N45, N70, N71, N72, N73, N74, N76, N770, N771, O080, O23, O85, O86, O98, T835, T836
Skin and soft tissue	A184, A422, A431, A46, A513, B35, B36, B372, B383, B403, B430, B432, B452, B463, L00, L01, L02, L03, L04, L05, L09, L303, T20, T21, T22, T23, T24, T25, T26, T813
Central nervous system	A17, A390, B375, B384, B431, B451, G00, G01, G02, G042, G050
Endocarditis/Circulatory	A395, A520, B376, I30, I31, I32, I33, I40, I520, T826
Musculoskeletal system	A180, A544, B453, M00, M01, M02, M03, M462, M463, M465, M490, M491, M492, M493, M60, M631, M650, M651, M710, M711, M730, M731, M86, M900, M901, M902, T845, T846

Table S2. Codes for focus of comorbidities

Congestive heart failure	I500, I501, I509
Chronic pulmonary disease	J430, J431, J432, J438, J439, J440, J441, J448, J449, J450, J451, J458, J459, J46, J47
Diabetes mellitus	E10, E11, E12, E13, E135, E136, E137, E139, E14, E831, E881, E888, E891
Chronic renal failure	E102, E112, E142, I120, N185, N189, N19
Liver disease	B181, B182, E106, E116, E146, K703, K704, K709, K711, K717, K720, K721, K729, K743, K744, K745, K746, K761, K766, K769, P788
Cerebrovascular disease	I60, I61, I63, I64, I690, I691, I693, I694, G409, G819

Solid cancer	C00, C01, C02, C03, C04, C05, C06, C07, C08, C09, C10, C11, C12, C13, C14, C15, C16, C17, C18, C19, C20, C21, C22, C23, C24, C25, C30, C31, C32, C33, C34, C37, C38, C410, C411, C44, C482, C50, C510, C511, C512, C519, C52, C530, C531, C538, C539, C540, C541, C543, C549, C55, C56, C570, C579, C58, C600, C601, C602, C609, C61, C629, C630, C632, C637, C639, C64, C65, C66, C670, C671, C672, C673, C674, C675, C676, C677, C679, C680, C73, C740, C749, C750, C760, C761, C780, C782, C783, C784, C786, C787, C791, C792, C793, C794, C795, C795, C796, C798, C799, C800, C809, D000, D001, D002, D010, D011, D012, D013, D014, D019, D020, D021, D022, D023, D040, D041
Non-solid cancer	B211, B212, C792, C793, C795, C810, C811, C812, C813, C814, C817, C819, C820, C821, C823, C824, C826, C827, C829, C830, C831, C833, C835, C837, C838, C844, C846, C847, C848, C851, C852, C859, C860, C861, C862, C863, C865, C866, C884, C901, C910, C911, C913, C914, C915, C916, C917, C918, C919, C920, C921, C922, C924, C925, C927, C928, C929, C930, C931, C933, C939, C940, C942, C943, C947, C950, C951, C959, D043, D044, D045, D046, D047, D049, D050, D051, D059, D060, D061, D069, D070, D071, D072, D073, D074, D075, D090, D092, D099, D471, D475, D648, D728, D763, H350, L270, P002, P358, Q859, R522, T451, T889, Z080, Z120, Z121, Z122, Z123, Z124, Z125, Z126, Z988

Table S3. ICD-10 and Japanese procedure claims codes for organ dysfunction

Type of organ dysfunction	ICD-10 codes	Type of procedure codes
Cardiovascular	I95, R57	Vasopressor use (Dopamine, Epinephrine, Norepinephrine, or Vasopressin)
Respiratory	J045	Mechanical ventilation
Neurologic	F05, G934	
Hematologic	D65, D695, D696, D698, D699	
Hepatic	K720, K763	
Renal	N17, J038	Renal replacement therapy

Table S4. Changes in the number of hospitals subject to DPC system

Year/ the number of hospital beds	<100	≧100 and <200	≧200 and <300	≧400 and <400	≧400 and <500	≧500 and <600	Summary
2010	156	288	282	244	149	271	1,390
2011	170	314	293	251	150	271	1,449
2012	181	335	301	265	149	274	1,505
2013	179	338	304	252	153	270	1,496
2014	200	373	318	262	155	277	1,585
2015	215	381	302	263	151	268	1,580
2016	243	420	314	272	152	266	1,667
2017	266	434	309	245	150	260	1,664
2018	303	463	317	244	147	256	1,730
2019	317	465	310	242	140	253	1,727

Figure S1. Annual changes in hospitalization by infectious disease and in-hospital mortality.

A, proportion of hospitalization in community-acquired infections by age subgroups (≤64 years: -1.63%/year [95%CI; -1.77%– -1.48%], adjusted R^2 = 0.99, P< 0.001; 65-74 years: +0.26%/year [95%CI; 0.11%–0.41%], adjusted R^2 = 0.63, P= 0.0037; ≥75 years: +1.37%/year [95%CI; 1.28%–1.45%], adjusted R^2 = 0.99, P< 0.001); B, proportion of hospitalization in nosocomial infections by age subgroups (≤64 years: -0.95%/year [95%CI; -1.13%– -0.76%], adjusted R^2 = 0.94, P< 0.001; 65-74 years: P= 0.95; ≥75 years: +0.94%/year [95%CI; 0.67%–1.20%], adjusted R^2 = 0.88, P< 0.001); C, community-acquired infections by age subgroups (≤64 years: -0.15%/year [95%CI; -0.25%– -0.05%], adjusted R^2 = 0.56, P= 0.075; 65-74 years: -0.89%/year [95%CI; -1.24%– -0.54%], adjusted R^2 = 0.79, P< 0.001; ≥75 years: -1.05%/year [95%CI; -1.45%– -0.64%], adjusted R^2 = 0.80, P< 0.001); D, nosocomial infections by age subgroups (≤64 years: -0.44%/year [95%CI; -0.64%– -0.24%], adjusted R^2 = 0.73, P= 0.001; 65-74 years: -0.86%/year [95%CI; -1.23%– -0.48%], adjusted R^2 = 0.75, P= 0.001; ≥75 years: -0.97%/year [95%CI; -1.38%– -0.55%], adjusted R^2 = 0.75, P< 0.001)

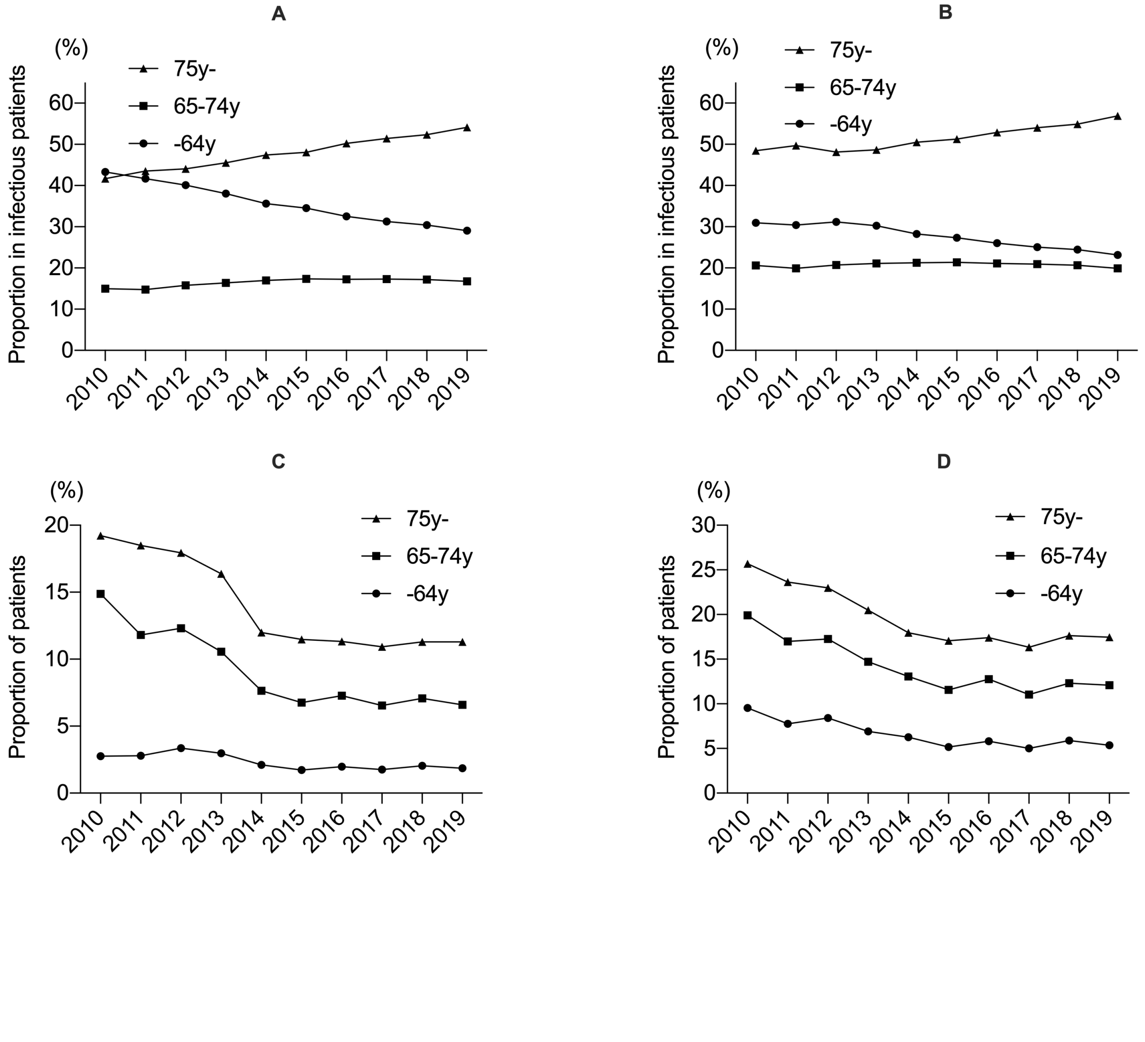

Figure S2. Proportion of focus in hospitalized patients by infection.

A, community-acquired infections; B, nosocomial infections.

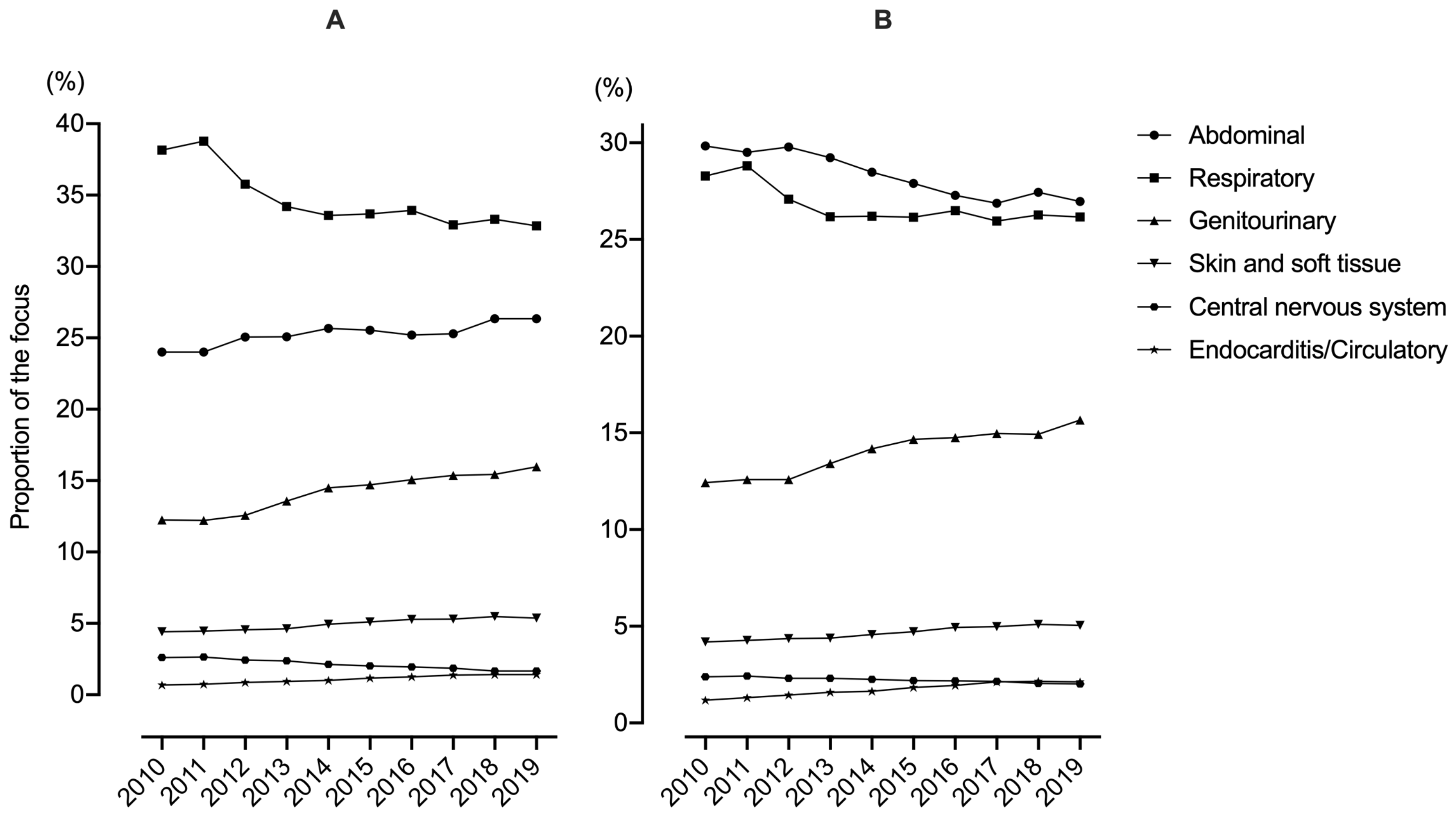

Figure S3. Trends in in-hospital mortality on each focus

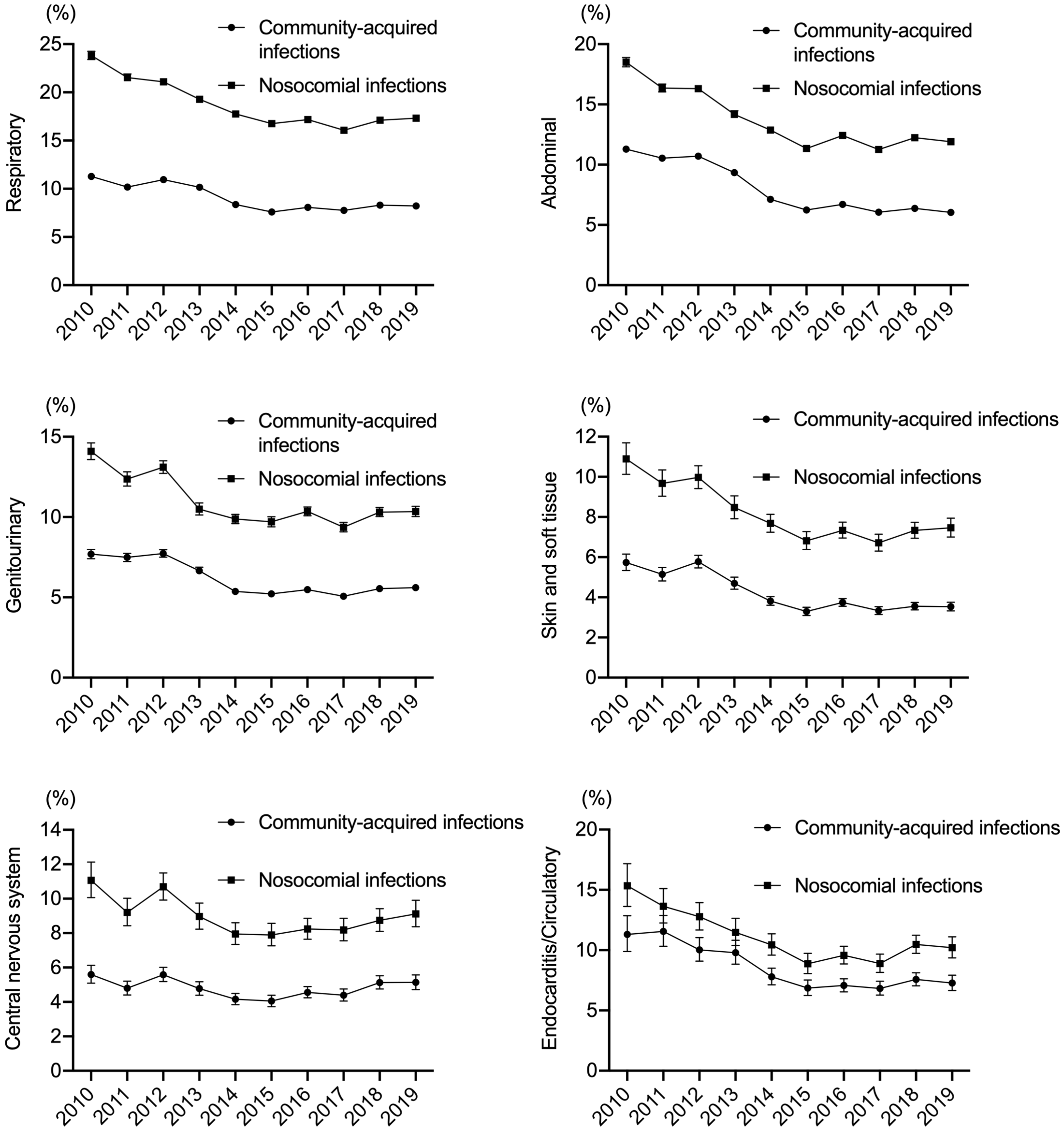

Supplement: Supplementary file 1 — Supplementary Material 1. [file 12879_2024_9353_MOESM1_ESM.pdf]
